# Superstitions of composure: the Ayn Rand cult and the pop-psychology of self-esteem

**DOI:** 10.1192/bjb.2024.17

**Published:** 2025-06

**Authors:** Marie Kolkenbrock

**Affiliations:** King's College London, London, UK

**Keywords:** Ethics, 20th century, philosophy, history of ‘self-esteem’, clinical malpractice

## Abstract

Ayn Rand is known as an advocate of rugged individualism and unregulated capitalism, which has led to a scholarly focus on her influence on neoliberal and right-wing politics. This article focuses on the psychologically unrealistic conceptualisation of self-esteem in Rand's ethics, which arguably prevails in today's self-help culture. Rand endorsed Nathaniel Branden, her acolyte and lover, as official therapist for her circle. In this role, he promoted the positive effects of living according to Randian principles on mental health. Rand's so-called objectivism therefore provides not only a questionable philosophical framework for neoliberal politics but also, and perhaps predominantly for its followers, a set of guidelines for the project of self-optimisation. The fact that Rand's ideal of radical self-sufficiency is ultimately psychologically unliveable makes its use in applied psychotherapy ineffective and harmful. The article offers a cultural-historical case study about the ideological entanglements of philosophy and pop-psychological concepts and of clinical malpractice.

Ayn Rand (1905–1982) is known as an advocate of rugged individualism and laissez-faire capitalism, which makes her a popular figurehead for proponents of libertarian and right-wing politics. The success of her writing is a compelling cultural phenomenon. Although Rand's rigour and talent as philosopher and literary author has been called into question from the beginning, the wider cultural currency of her ideas is undeniable: citing the consistently high numbers of sold copies as well as several high-profile republican politicians who have proclaimed their admiration for Rand's novels, American studies scholar Claudia Franziska Brühwiler claims that Rand is ‘undoubtedly one of the most politically influential fiction writers of the twentieth century’ (p. 2).^1^ Rand's popularity in conservative circles has led to a scholarly focus on the ideological tangents that grow from her work to the politics of neoliberalism as well as the ‘culture of greed’ or ‘age of selfishness’ that supposedly contributed to the 2008 financial crisis.^2,3^ What interests me for the purpose of this article, however, is the psychologically unrealistic conceptualisation of self-esteem that lies at the heart of Rand's ethics and that arguably prevails in some of today's self-help culture.

Rand defines her ethics, which she called objectivism, as a ‘morality of *rational* self-interest – or of *rational selfishness*’ (p. xi).^4^ Although this does not mean that she advocates for an unregulated pursuit of one's individual *desires*, as these can be irrational, one of the basic premises of Randian philosophy is that ‘man *must be the beneficiary of his own moral actions*’ (p. x).^4^ Thus, acting on behalf of another is seen as immoral:

‘The basic social principle of the Objectivist ethics is that just as life is an end in itself, so every human being is an end in himself, not the means to the ends or the welfare of others – and, therefore, man must live for his own sake, neither sacrificing himself to others nor sacrificing others to himself’ (p. 30).^4^

Although the last part of the sentence seems hardly compatible with the exploitative structures of unregulated capitalism, at a social and political level, this principle leads to the dismissal of the welfare state for which Rand is known and, in certain political milieus, admired. Not surprisingly, then, taxes, social work and state-funded community projects are all met with derision in Rand's work.

Yet more than in Rand's political and philosophical ideas themselves, her reach lies in the ability to infuse readers with lust and desire for aspiration, self-actualisation and control, argues American cultural studies scholar Lisa Duggan.^2^ And Rand biographer Jennifer Burns writes ‘for all her emphasis on reason it is the emotional and psychological sides of her novels that make them timeless’ (p. 286).^5^ Ironically, then, despite perpetuating an ideal of extreme rationality and emotional detachment, Rand's work contributes to the affective investments in neoliberal and individualist ideals. This recognition of the psychological dimension of Rand's work is helpful for the understanding of the intellectual and personal entanglements between the Randian school of objectivism and the popularisation of the psychological concept of ‘self-esteem’.

## Self-esteem in the philosophy of objectivism

The term ‘self-esteem’ occurs in Rand's own work with great frequency. In fact, according to John Galt, protagonist in her *magnum opus*, the novel *Atlas Shrugged* (1957), it makes up one of the three things that man must hold ‘as the supreme and ruling values of his life: Reason – Purpose – Self-Esteem’ (p. 1018).^6^ And any attempt at challenging her definition of ‘rational selfishness’ is an ‘attack on man's self-esteem’ (p. xi).^4^ Rand was notoriously uninterested in classical psychological questions, for example how someone's family relationships, personal history and inner life play a role in their personality and experience of the world: ‘When I'm questioned about myself, I am tempted to say, paraphrasing Roark [protagonist of her break-through novel *The Fountainhead* (1943)]: “Don't ask me about my family, my childhood, my friends or my feelings. Ask me about the things I think”’ (p. 669) .^7^ And yet, the centrality of self-esteem in her writing explicitly links her ethics to psychological health.

Psychology graduate Nathaniel Branden (1930–2014), Rand's acolyte and (for a period) lover, was then responsible for promoting this link, i.e. the positive effects of living according to objectivist principles on mental well-being. These efforts did not remain at a theoretical level. Nathaniel and his first wife Barbara started a lecture series on ‘The Basic Principles of Objectivism’. This series became so successful that it formed the foundation for a full-fledged educational enterprise, the Nathaniel Branden Institute (NBI), founded in 1958. Rand saw Nathaniel as her intellectual heir. ‘She had described me as its [her philosophy's] most consistent embodiment,’ recalls Branden modestly in his memoir (p. 1).^8^ Of course, this character evaluation would change later when Branden rejected Rand's attempts to reignite their sexual affair and when she found out that he had begun another affair with a third, younger, woman, who would later become his second wife. This relational split led to the dismantling of the NBI in 1968, but in the early years after its foundation, Branden had Rand's full support.

Branden's practice as an (unlicensed) psychotherapist directly benefited from this close affiliation with Rand: ‘he had already begun to establish himself as a therapist on Rand's coattails, drawing patients primarily from those who found her work interesting. Now he started a second business [the NBI] drawing on Rand's ideas’ (p. 180).^5^ And this second business fed back into the first: ‘Nathan and [his cousin, the psychiatrist] Allan Blumenthal received the bulk of their therapy clients from NBI students’ (p. 215).^5^ Rand ‘endorsed Nathaniel Branden as official therapist to everyone in [her circle], to root out irrationalities incompatible with the perfect Objectivist character expected of those closest to Rand. In imitation of this model, practically everyone in the wider circle of New York City Objectivists was soon seeing an Objectivist psychotherapist’ (p. 133) – which meant Nathan himself, his cousin Allan Blumenthal or someone sanctioned by either of them.^9^ The promise of seeing an objectivist therapist was to ‘get your premises straightened out’ (p. 134),^9^ recalls journalist Joan Kennedy Taylor, an early member of the objectivist movement.

But what exactly did these premises entail? In combination with their great talent and physical attractiveness, it is their indifference to approval from others that makes Rand's heroes inviting identificatory characters for narcissistic fantasies of self-actualisation. Seeking affirmation from others is unempathetically described as a moral failing that stands in the way of such self-actualisation: ‘The misery of knowing how strong and able one is in one's own mind, the radiant picture never to be made real’, comments the narrator in *The Fountainhead*, ‘Dreams? Self-delusion? Or a murdered reality, unborn, killed by that corroding emotion without name – fear – need – dependence – hatred?’ (p. 709).^10^ Of course, the protagonist Roark is free of that kind of ‘chronic [ … ] fear, in which they all [i.e. the common people] lived’ (p. 709)^10^ and which apparently stands in the way of their actualisation of that radiant picture of themselves. It is the absence of this fear that enables Roark to achieve real greatness and untainted self-esteem. Rand's philosophy therefore not only provides an ethical framework in favour of laissez-faire capitalism but also offers a set of guidelines for the project of self-optimisation according to objectivist principles, which was then further developed by Branden.

## Radical self-sufficiency

One of Branden's essays may give some insight into how the fear to which Rand's narrator refers relates to her understanding of self-esteem. In the essay ‘Mental health versus mysticism and self-sacrifice’ (1963), he proposes that ‘self-esteem’ is the essential prerequisite to living a life ‘committed to reason’, while anxiety and guilt are the ‘antipodes of self-esteem and the insignia of mental illness’ (p. 41).^11^ It is certainly not coincidental that, in classic psychoanalysis, both anxiety and guilt are seen to occur predominantly relationally: as functions of the superego, they keep the individual's aggressive and antisocial impulses in check. The fear that Rand's narrator condemns sounds much like the ‘social anxiety’ that Sigmund Freud described as a ‘fear of loss of love’ (p. 124).^12^ For Freud, as well as for attachment theorists following in his wake, this attachment to the other is not a choice but a necessary element of subject formation. Of course, the process of growing up involves the individuation of the self, but this does not mean a complete severance of attachment: in the words of the Scottish psychiatrist and psychoanalyst W. Ronald D. Fairbairn, we move from ‘infantile dependency’ to ‘mature dependency’ (p. 40), which is a mutual, reciprocal form of other-reliance.^13^ Psychoanalytically speaking, the superego continues to keep us in check through feelings of guilt and anxiety. Although these can certainly take on pathological forms, they are also just part of what it means to exists as an individual in society.

However, in Rand's philosophy and in Branden's psychological interpretation of it, such attachment to and dependency on others are presented as restrictive and as signs of a lack of integrity. Of course, it is undeniable that relying all too heavily on the approval of others for one's sense of self will be detrimental to a one's self-esteem and experienced as suffering. However, Rand's ‘ideal man’ is an ‘end in himself’ in the absolute sense. A Randian hero has no need for other people other than in their function to facilitate the realisation of his inherent greatness. ‘I need people to give me work’, says the architect Howard Roark in *The Fountainhead*. ‘Do you suppose I should need them in some other way? In a closer, more personal way?’ And when his conversation partner clarifies: ‘You don't need anyone in a very personal way’, Roark confirms this with an unequivocal ‘No’ (p. 158).^10^ With this disinterest in interpersonal connection, Roark represents a psychological ideal of radical emotional detachment. His indifference to the needs and feelings of others includes their perception of himself: he does not care what others think of him. This idealised lack of attachment is made more poignant through the character of Peter Keating, whose relationship to his overbearing mother is only one example of his dependency on others and the external validation they offer. As a prototypical ‘social metaphysician’, for whom the ‘moral appraisal of himself by others is a primary concern’ (p. 165), he therefore functions as a contrast to Roark's exceptional self-sufficiency.^14^

All of Rand's protagonists cultivate this kind of interpersonal distance. Dagny Taggart, the heroine in *Atlas Shrugged*, notes ‘that the sense of detachment one feels when looking at the earth from a plane was the same sense she felt when looking at people: only her distance from people seemed longer’ (p. 832).^6^ This detachment enables Rand's heroes to strive for their goals without the distractions and demands that relational bonds tend to involve. Rand herself, however, had the painful experience of not living up to her own psychological ideal of emotional detachment and unshakeable self-esteem. When the reception of *Atlas Shrugged* did not live up to Rand's over-ambitious expectations, Branden insists that she was initially a picture of an ideal objectivist character: she ‘acted equally indifferent to praise and criticism’ and ‘conveyed a stony calm of someone who is settling in for a long siege’ (p. 203).^8^ However, after a while, she slipped into a deep depression. Realising that this was not compatible with the ideal of emotional detachment and self-sufficiency she had formulated in her novels, she said: ‘Galt [the protagonist of *Atlas*] would handle all this differently. Somehow, he would be more untouched by it. I would hate for him to see me like this’ (p. 213).^8^ Rand's own case therefore shows that remaining perpetually unaffected by the other is neither healthy nor possible.

## Clinical malpractice

The extreme ideal of self-reliance we find in Rand's work perpetuates what psychoanalyst Adam Phillips has called a ‘superstition of confidence in the integrity of the self’ (p. 42).^15^ This superstition materialises in an extreme form of emotional composure:

‘There is a familiar type of composure that creates an appearance of self-possession [ … ]. The mind creates a distance in the self [ … ] from its own desire, from the affective core of the self, and manages, by the same token, a distance from everybody else. [ … ] Hell is not other people but one's need for other people’ (p. 45).^15^

By terming it a ‘superstition’, Phillips makes clear that this kind of radical self-sufficiency is not a realistic – or even desirable – mode of being.

A psychotherapeutic approach based on the unrealistic objectivist psychological ideal would therefore be ineffective at best and extremely harmful at worst. This was recognised by psychologist Albert Ellis, founder of the rational emotive behaviour therapy (REBT) approach. He was keen to stress that despite ‘many superficial resemblances’ to his practice, ‘objectivist teachings are unrealistic, dogmatic, and religious, that [ … ] they are likely to create more harm than good [ … ], and that they result in a system of psychotherapy that is inefficient and unhelpful’ (p. 11).^16^ Judging from Walker's compilation of multiple examples of clinical malpractice by objectivist therapists, this was precisely the outcome of many of the psychotherapeutic treatments carried out in objectivist circles in the 1960s, ’70s, and ’80s.^9^ From Branden treating (and gaslighting) his own wife to Blumenthal protégé Lonnie Leonard legitimising his sexual abuse of his patients with objectivist principles, insiders seem to recall objectivism as a therapeutic cult that perpetuated a culture of fear, judgement and exploitation.

Branden, after his break with Rand, went on to develop the objectivist notion of self-esteem into his own pop-psychological brand. Between 1969 and 1998, he wrote more than a dozen self-help books, all concerned with the themes of self-realisation and self-esteem. While he distanced himself from Rand as a person, he tacitly retained many of her objectivist premises. In *The Six Pillars of Self-Esteem* (1994), one of his last books, he writes: ‘*Never ask someone to act against his or her self-interest as he or she understands it*’ (p. 113).^17^ In a very Randian manner, he argues that ‘teaching cognitive skills, the values of works ethic, self-responsibility, interpersonal competence, the pride of ownership’ is of utmost importance in the fight against poverty, because the ‘philosophy of victimhood has not worked’ (p. 298).^17^ And the way he bemoans the alleged cultural tendency to value ‘self-sacrifice’ more than ‘intelligent selfishness’^17^ (p. 121) could be taken straight out of *The Fountainhead* or *Atlas Shrugged.* Branden modified Rand's ideal of absolute self-sufficiency, but the extent to which the book preaches self-reliance still rings extreme: ‘No one is coming’ was one of the motivational quotes he used in his therapy groups to inspire self-responsibility in his clients.^17^ Not surprisingly, Branden's notion of self-esteem ‘represents in some ways the exact reverse’ of the concept of self-esteem ‘which became influential in education and elsewhere’ and which de-coupled praise and rewards from achievement (p. 166).^9^ Doubtlessly, he nevertheless both benefited from and contributed to the growing popularity of the concept.

Two things are perhaps particularly insidious about a mental health provider like Branden embracing and implementing the ethics of objectivism in his practice. First, by constructing a psychological ideal that was ultimately unachievable, the objectivist movement *created* a need for self-improvement and therefore psychotherapeutic treatment in its followers. Second, since objectivism was, in the first instance, an ethical framework, failure to live up to its ideals was implicitly equated with moral deficiency – and, logically, so was any refusal to get treated. Suffering from low self-esteem became then not only a matter of mental well-being but also a sign of failure to embrace objectivist premises. Combined with the rejection of the welfare state and an alleged ‘victim culture’, Rand and Branden represent a particularly brutal understanding of self-esteem: it makes people individually and exclusively responsible for their mental health and demeans people who are suffering from poor self-esteem and other mental health problems, such as depression and anxiety. In this way, Rand's and Branden's conduct code of composure aligns with what Gary W. Wood has identified as the ‘guru-ization of self-help’.^18^ As part of this guru-ization, ‘impossible objectives’ create a certain double-bind by linking self-esteem to ‘unrealistic “change your life” goals’. Indeed, objectivism aligns with other dominant self-improvement trends through the idea that ‘we have the power to control our lives if only we knew how to harness it’.^19^

## Self-optimisation in the age of *Homo economicus*

The idea that we are in complete control of – and therefore solely responsible for – our lives is one of the central myths that are frequently perpetuated in self-help books.^18^ This myth serves policies that reduce state-funded poverty and mental healthcare projects. It is therefore perhaps not surprising that the libertarian anti-psychiatrist Thomas Szasz, although explicitly not a fan of either Rand or Branden, lauded Rand for rejecting depression as a medical issue.^20^ The ironic and unlikely alignment of the antipsychiatry movement's revolutionary goals of abolishing asylums with the politics of austerity that promoted the decimation of state-funded mental healthcare provision, as criticised by Peter Sedgwick in his 1982 book *Psychopolitics*, becomes palpable in Szasz's affirmative nod to Rand.^21^

The connection between the emphasis on self-responsibility and rationality on one hand and the propagation of unregulated capitalism on the other has been well-recognised beyond the context of Rand's work, of course: in a society increasingly oriented towards market values, ‘the self is driven to constantly improve, change and adapt’ and becomes an ‘entrepreneurial self’ (back cover).^22^ In terms of the primary emphasis on rational self-interest, Rand's ideal man corresponds to the figure of *Homo economicus* that provides the model for the capitalist subject guided by utility and optimisation.^23^ For Giovanni Stanghellini, the rise of *Homo economicus* in capitalist culture has transformed narcissism ‘from a niche phenomenon’ and a ‘psychopathological category’ into a ‘mass phenomenon’ and an ‘idealized model’.^23^ Stanghellini abstains from the kind of reactionary cultural pessimism one can find in Christopher Lasch's 1979 diagnosis of 20th-century US-American culture as a ‘culture of narcissism’:^24^ without the underlying nostalgia for the traditional patriarchal family palpable in Lasch's writing, Stanghellini characterises the narcissism of *Homo economicus* as an inability to tolerate the other in their otherness. The other is not allowed to exist in separation, but must either be consumed and appropriated or, if that proves impossible, annihilated. In other words, others either serve us or stand in our way. The manner in which *Fountainhead* protagonist Roark limits his need for others to the ways they can facilitate the realisation of his architectural projects provides a good example of how Rand's work aligns with the foundational values of *Homo economicus*.^23^

What separates Rand's heroes from the aseptic character of *Homo economicus*, however, is that passion and desire are driving features of their character: this is because they are masters in the practice of delayed gratification. Where *Homo economicus*, as described by Stanghellini, has no tolerance for the unpleasant feeling of not-having, Rand's heroes and heroines will always take the promise of reaching perfection in a distant future over the offer of temporary comfort or enjoyment in the here and now. This preserves the element of distance that is absent from the world of the *Homo economicus*, in which both the distance necessary for desire and the closeness necessary for intimacy are replaced by consumer-ready proximity.^23^ One may assume that it is this element of passion that infuses Rand's work with its affective appeal.

Ultimately, the Randian promise of a dormant ‘radiant picture’ of the self that could come to life if only we stopped sacrificing our own self-interest to please others is the phantasy of a Rogerian actualising function that is immune to any forms of ‘social mediation’.^25^ It is this seductive phantasy, I believe, that becomes easily exploitable through the ‘guru-ization’ of self-help.

## Randian ‘immunity’ in self-help culture

There is nothing wrong with the wish to care less about external approval and to lead a more self-directed life. Numerous self-help books today are dedicated to this goal, and some of them will undoubtedly be helpful to individuals. It is therefore not my aim to discount the potential of self-help or self-directed ‘bibliotherapy’ to improve aspects of peoples’ lives. Moreover, strengthening the ability to ‘not care’ may be particularly beneficial for people socialised as women, who, based on patriarchal gender roles, are more likely to have been conditioned to subordinate their own self-interest to the care for others.^26^ And finally, the understanding that humans have the ability and often the desire to ‘improve’ themselves does not automatically lead to a Randian dismissal of care for others. As cultural historian and life coach Anna Katharina Schaffner puts it, ‘the point of all worthwhile self-improvement’ is not inward-looking optimisation, but to ‘free up our energies so that we can direct them toward other people and toward creative projects’ (p. x).^27^

However, the superstition of composure in Rand's books as well as their ongoing success, I think, teach us something about a desire for what the Italian philosopher Roberto Esposito calls ‘immunity’: a sense of being ‘immune’ to the demands and restrictions that the shared obligations of living in community with others put on us (p. 49).^28^ Branden makes this immunitary logic in his understanding of self-esteem unequivocally clear by calling it ‘the immune system of consciousness’ (p. 3).^17^ ‘In modern societies,’ he writes further, ‘[the thrust towards self-realisation] is also associated with political freedom, with the liberation of humankind from servitude from one tribe or another’ (p. 124).^17^ Certainly, Branden softens the Randian individualism by stating that the two complementary tracks for successful self-esteem are that of individuation and that of relationship. And yet, his book does not tell us much about the ambivalence and frustration that are necessary parts of any relational dynamic.

Perhaps the important fact that self-help books (as well as life-coaching social media accounts and YouTube channels) cannot provide a ‘therapeutic relationship, which is a core factor in the healing process’,^18^ creates an entryway for Randian superstitions of composure. This becomes potentially insidious when this lack of the therapeutic relationship is concealed through the para-social attachment that can be formed to the public persona of the author or other self-help gurus on social media. Certainly, the genre as a whole is not guilty of promoting such superstitions, but it may be fair to say that the genre lends itself to those who want to promote them. This is more likely when the aim of a self-help provider is not to inform but to ‘entertain or to sell’.^18^

It is not news that trends of self-improvement can easily be recruited to do, as it were, the dirty work for neoliberal systems by placing responsibility for failure and success exclusively on the individual. Consequently, they can undermine struggles for collective empathy, solidarity and care. Although Rand's work is not the only ideological origin for this hyper-individualistic thinking, the cultural history of objectivism provides an exceptionally clear case study of the exploitative potential of the self-improvement industry. And in view of the therapeutic cult that formed around Rand's and Branden's personas, their case study makes us aware of how easily the promise of better mental health can be used to serve wider ideological agendas. It seems vital for mental health practitioners to be critically aware of these trends and the (pseudo-)clinical malpractice they may inspire.

## Data Availability

Data availability is not applicable to this article as no new data were created or analysed in this study.

## About the author

**Marie Kolkenbrock** holds a PhD in German studies from the University of Cambridge (2014) and is a Branco Weiss Fellow in the Department of Languages, Literatures and Cultures at King's College London, London, UK.
